# *Zanthoxylum armatum* DC extracts from fruit, bark and leaf induce hypolipidemic and hypoglycemic effects in mice- in vivo and in vitro study

**DOI:** 10.1186/s12906-018-2138-4

**Published:** 2018-02-20

**Authors:** Fiaz Alam, Qazi Najam us Saqib, Mohammad Ashraf

**Affiliations:** 1 Department of Pharmacy, COMSATS Institute of Information Technology, Abbottabad, -22060 Pakistan; 2 Department of Biochemistry and Biotechnology, The Islamia University of Bahawalpur, Bahawalpur, -63100 Pakistan

**Keywords:** *Zanthoxylum Armatum*, Antidiabetic, Biochemical parameters, α-glucosidase inhibition

## Abstract

**Background:**

*Zanthoxylum armatum* DC is an important medicinal plant of south East Asia, and has been used to treat various ailments in traditional medicine including diabetes. This study investigated the in vitro and in vivo antidiabetic and biochemical effects of extracts of *Z. armatum* in mice.

**Method:**

The extracts of fruit, bark and leaf from *Z. armatum* were tested for α-glucosidase inhibition activity. Albino mice of either sex weighing (26–30 g) assigned into groups. Diabetes was induced by IP injection of alloxan monohydrate (150 mg/kg). The extracts (500 mg/kg) and standard (Glibenclamide 10 mg/kg) were administered to mice for 15 days. Serum biochemical parameters were monitored for the period of study.

**Results:**

The leaves and bark extracts showed maximum α-glucosidase inhibition (96.61 ± 2.13 and 93.58 ± 2.31% respectively). The extracts treated and the standard treated groups showed significant decrease in the fasting blood glucose levels compared to diabetic control. The effect was more pronounced in mice treated with leaves extract. In the in vivo studies body weights of diabetic mice treated with *Z. armatum* extracts and the standard did not reduced to extent as observed in diabetic control and this difference was significant (*p* < 0.05). There was a significance (*p* < 0.001) improvement in blood hemoglobin, urea, creatinine, cholesterol, and triglycerides of the extracts treated diabetic mice. The extracts showed hypolipidemic effect by reducing the LDL level. The extracts produced no prominent changes in proteins levels.

**Conclusion:**

It can be concluded that *Z. armatum* extracts showed excellent antidiabetic potential in vivo and in vitro and could be considered for further appraisal in clinical assessment and drug development.

## Background

Natural products and their derivatives have been successful source of bioactive molecules in medicines much before the advancement of other modern therapeutics in the post-genomic era [1]. Studies conducted in several developed countries reported that almost half to two thirds of the population affected with diabetes use complementary and alternative medicine to control the condition [2]. The world health organization has recommended and encouraged the use of alternative therapy especially in countries where access to the conventional treatment of diabetes is not adequate [3].

Medicinal herbs are expected to have a similar degree of efficacy without the troublesome side effects associated with conventional drug treatment [4]. A multitude of herbs and medicinal plants and some compounds purified from them have been studied for the treatment of diabetes throughout the world as they might provide a basis of new synthetic antidiabetic analogues with potent activity [5]. Plants which have been shown to have hypoglycemic action, act on blood glucose through different mechanisms. Some of them may inhibit endogenous glucose production [6] or interfere with gastrointestinal glucose absorption [7] and some may have insulin-like substances [8]. World ethnobotanical information about medicinal plants reports almost 800 plants used in the control of diabetes mellitus [9]. There is much need to explore such resources for the development of new medicines to control or treat diabetes.

*Zanthoxylum armatum* DC is a small tree almost entirely glabrous with a strong pungent and aromatic smell. Its local name is Timbar or timar (in Hindko), Tejmal, Nepali dhania (in Urdu). It is found in hot valleys of subtropical Himalaya. In Pakistan it is found in Dir, Swat, Hazara, Murree and Poonch hills and in Jhelum. The seeds and bark are used as an aromatic tonic in fever, dyspepsia and in cholera. The fruit as well as branches and thorn are used as remedy for toothache, also as stomachic and carminative and employed as fish toxin [10]. Various parts of this *Z. armatum* are used in the preparation of tooth powder and medicinal preparations. The studies on this plant during the last few decades show that these plants contain various useful pharmacological active compounds [11].

The evidences are available where *Z. armatum* leaves water extract was tested for anti-diabetic potential in animals. The experiments demonstrated that *Z. armatum* water extract possess anti-diabetic activity in in-vivo procedure using mice [12]. Similarly, in another experiment the hydromethanolic extract of bark of *Z. armatum* was evaluated for its antidiabetic activity in streptozocin-induced diabetes in rats. The total cholesterol, triglycerides, low density lipoprotein, very low density lipoprotein were also monitored [13]. So, there are enough evidence available for testing *Z. armatum* for anti-diabetic potentials in animal models. Keeping in view the importance of this plant genus in management of diabetes this work was carried out to investigate the potential of *Z. armatum* against diabetes.

## Methods

### Chemicals

Chemicals used in experiments were of analytical with high purity grade procured from standard commercial sources. Organic solvents: Methanol (CAS No. 67–56-1.), Diethyl ether (CAS 60–29-7), Ethanol (CAS No. 64–17-5), Ethyl acetate (CAS 141–78-6), Chloroform (CAS 67–66-3), n-Hexane (CAS 110–54-3) from Merck (Germany). Glibenclamide (CAS Number: 10238–21-8), Alloxan monohydrate (CAS Number: 2244–11-3) from Sigma Aldrich (CAS Number: 2244–11-3) from Fluka chemicals. Glucose 5% Normal Saline, 0.9% from Shahzeb Pharma, Pakistan. Cholesterol kit, Triglycerides kit, Hb Kit from Erba. Acarbose (CAS Number: 56180–94-0), a-glucosidase (CAS Number: 9001–42-7), Sigma–Aldrich Co., St. Louis, USA.

### Instruments

Feeding Tube Syringes/butterfly needle from Pharmax, (Pakistan). Weighing balance from Sartorius (GE412 scale). Glucometer from Accucheck (Model Aviva by Roche, Germany). Cylomixer (CM 101 plus) from Remi (India). Rotary evaporator Laborota 4000 from Heidolph (Germany).

### Plant material

Five kg of each of leaves, bark and fruit of *Z. armatum* were collected form Tanawal area of KPK Pakistan in the month of August, 2013. After authentication from plant taxonomist Manzoor Hussain and specimen voucher (PB025) was deposited in the herbarium of the Post graduate college, Abbottabad. Each part of the plant was washed under running water and dried in shade at room temperature and was ground to coarse powder. The powder drug was stored in air tight and light resistant container before extraction.

### Preparation of plant extracts

The powder material (100 g) of the fruit, bark and leaves was extracted with methanol using soxhlet extractor for 20 h each. It was filtered through a Whatman Grade-I filter paper. The filtrate was evaporated on a vacuum rotary evaporator under reduced pressure at 40 °C. The desiccator was used to remove the remaining moisture, and finally the extracts were stored in air tight containers at 4 °C for further use.

### Experimental animals

Healthy adult albino mice (26–30 g) of either sex were selected for the study. The animals were obtained from National Institute of Health (NIH) and then bred in Animal house of CIIT Abbottabad. Mice were housed in polypropylene cages (47 × 34 × 20 cm) lined with husk (renewed every 24 h). They were given a standard diet and water ad libitum. The pellet diet consisted of 23% protein, 5% lipids, 4% crude fiber, 8% ash, 1% calcium, 0.6% phosphorus, 3.4% glucose, and 5% nitrogen-free extract (carbohydrates). After experiment the animals were euthanized by applying three times the dosage of pentobarbital through intraperitoneal injection. The approval number PHM-Eth/CF-M04/11–24 of the Research, Ethical Committee (REC), department of Pharmacy, CIIT, Abbottabad was taken before the animal studies were conducted.

### α-glucosidase inhibitory assay

The assay was carried out according to the method described by [14] with slight modification. All the samples were dissolved in DMSO. An enzyme solution containing α -glucosidase (0.8 units/ml) in 50 mM phosphate buffer with pH 7, containing 100 mM NaCl was made immediately before use. The solution was kept on ice during the experiment. The substrate, pNP-G (0.7 mM) in phosphate buffer, was prepared fresh before use. The test solution (20 μL) and enzyme solution (80 μL) was pre-incubated for 5 min at 37 °C. The reaction was initiated with 1.9 mL of substrate solution and incubated for fifteen minutes at 37 °C. The reaction was stopped by adding 2.0 mL (0.5 M) aqueous Tris solution, and the absorbance of PNP released from PNP-G was measured at 400 nm. 20 μL DMSO was kept as blank (Without addition of test solution). Acarbose was used as a + tive control. Analysis was carried out in triplicates, and the results were calculated as ±SEM.

Percent α-glucosidase inhibition was calculated as follows: (1–B/ A) × 100, where A is the absorbance of control and B is the absorbance of samples containing extracts.

### Oral glucose tolerance test

Before the induction of diabetes the oral glucose tolerance test was performed in overnight fasted (18 h.) normal mice as per [15]. Healthy mice were randomly selected and distributed into five groups (*n* = 6). Glucose (2 g/kg b.w.) was fed. Blood was taken out from the tail vein at 0, 60, 90, 120 and 150 min of glucose administration and glucose levels were estimated.

### Induction of diabetes and experimental design

Antidiabetic activity was carried out on selected healthy albino mice [16]. The experiments were carried out in accordance with the National Institute of Health guidelines of care and use of laboratory animals [17]. Diabetes was induced in mice using freshly prepared solution of alloxan monohydrate dissolved in normal saline (0.9% *w*/*v* of NaCl). For inducing diabetes, the mice were kept on fasting for 12 h and were given a single IP injection of alloxan monohydrate (150 mg/kg b. wt.). To prevent fatal hypoglycemia initially due to massive pancreatic insulin release, the mice were provided with 5% glucose solution after six hours supplied in water bottles in their cages for next 24 h. Animal were kept at room temperature (27 ± 2 °C) and humidity (55 ± 5%) and a 12 h’ cycle of light and dark. After 72 h, the glucose level of the fasting animals was measured. After acclimatization, the animals were separated into following groups (six mice in each group); Groups A, Normal control treated with saline; B, Diabetic control; C, Diabetic mice treated with 500 mg/kg body weight of fruit extract; D, Diabetic mice treated with 500 mg/kg body weight of bark extract; E, Diabetic mice treated with 500 mg/kg body weight of leaves extract F, Normal mice given 500 mg/kg of Gt-MeOH extract and G, reference control treated with glibenclamide (10 mg/ kg). An identification mark was given to the mice of each group on the tail with permanent marker. Each of mice was weighed and the doses were calculated accordingly. The extract was given orally. All the groups were given respective treatments daily for 15 days. To check the effect of the extracts on the weight of animals, weight of the mice was recorded prior to the administration of the extracts and at the end of the study as well i.e. on the 15th day.

The blood samples were collected (in glass tubes) and left for 1 h at 37 °C to allow to clot. The blood was collected using capillary tubes into Eppendorf Tubes® containing heparin for analysis of plasma profile. Using a glass Pasteur, carefully, the clot was loosened from the sides of the tube. The serum was centrifuged at 5000 rpm for 5 min at 4 °C. The serum was removed from the clot by gently pipetting off into a clean tube using a micropipette. The serum was labeled with the animal number and the estimations were made [18].

### Biochemical analysis

The blood sugar level was measured using Accu-Chek® Active test strips in Accu-Chek® Active test meter by collecting the blood from the vein of mice tail. Total cholesterol and triglycerides were assayed using the protocol of [19].The level of serum urea and creatinine were assayed using the protocol given by [20]. Total proteins were assayed using protocol described by [21]. HDL and LDL were measured by the protocol given by [22].

### Statistical analysis

All the values including body weight, fasting blood sugar, and biochemical estimations were expressed as mean ± standard deviation (S.D.) and analyzed for ANOVA –Dunnet’s test. Differences between groups were considered significant at *p* < 0.001 and *p* < 0.05 levels. The normal control was compared with the normal extract treated groups while diabetic control was compared with the diabetic extract treated and Glibenclamide treated groups.

## Results

### α-glucosidase inhibitory activity

The extracts Zf, Zb and Zl from *Z. armatum* showed significant inhibition of α-glucosidase enzyme in an in-vitro antidiabetic assay. The extracts Zl and Zb inhibited the enzyme with percentage and IC_50_ values of 96.61 ± 2.13 (IC_50_ = 47.87 ± 0.45) and 93.58 ± 2.31% (IC_50_ = 21.82 ± 0.87) respectively. The fruit extract also showed very good activity and inhibited the enzyme with percentage inhibition and IC_50_ values of 83.76 ± 3.01% (IC_50_ = 31.62 ± 0.67).

### Effect on body weight

The effect of extracts of *Z. armatum* on body weight of mice is shown in Table 1. The table for the body weight changes shows that there is significant increase in the body weight of the extract and standard treated groups when compared to the diabetic group (*p* < 0.05) over the period of 15 days. The diabetic group showed decrease in the body weight. The groups of normal mice treated with extracts were compared with diabetic control group for changes in body weights. The extracts of *Z. armatum fruit* (Zf), bark (Zb) and leaves (Zl) showed a significant increase in body weights (p < 0.05) of normal mice as compared with diabetic group treated with extract. However, leaves extract showed comparatively less effects on body weights of normal as well as diabetic treated groups.

**Table 1:** Effect of extracts of *Zanthoxylum armatum* and standard drug Glibenclamide on body weight of normal and alloxan monohydrate induced diabetic mice

Groups	Body weight in grams			
	Before treatment	After 7 days	After 15 days	% Variation
Normal	27.67 ± 2.3	30.83 ± 4.3	34.33 ± 6.3	10.77%
Diabetic control	32 ± 1.8	28 ± 3.0	25 ± 3.8**	12.39%
Normal Zf treated	29.67 ± 2.33	31.33 ± 2.50	33.83 ± 2.04**	6.62%
Diabetic Zf treated	31 ± 1.1	25 ± 1.8	26 ± 2.4	12.81%
Normal Zb. Treated	29.67 ± 2.33	31.00 ± 2.09	33.00 ± 2.09**	5.37%
Diabetic Zb treated	30.00 ± 2.82	28.83 ± 3.83	27.27 ± 3.06	4.77%
Normal Zl treated	30.00 ± 1.78	31.00 ± 1.67	32.83 ± 1.83	4.59%
Diabetic Zl treated	29.00 ± 2.75	21.67 ± 2.94	24.50 ± 1.04	14.96%
Diabetic Glibenclamide (10 mg/kg)treated	30 ± 1.8	30.01 ± 1.7	29.3 ± 1.8	13.39%

Data represented as mean ± S.D. values of 6 animals each *p < 0.001, **p < 0.05 (One wayANOVA, Dunnet’s t-test, Graph pad prism software). Normal control was compared with extract treated normal groups. The diabetic control was compared with extract treated diabetic groups and standard

### Effect of different extracts of *Zanthoxylum armatum* on blood glucose levels

The induction of diabetes has caused significant initial increase in the fasting blood glucose levels of all the groups. The diabetic control group shows significant increase throughout the study period as compared with the normal control group (*p* < 0.001). However, the extracts treated groups and the standard treated group shows significant decrease in the fasting blood glucose levels as compared with diabetic control which was determined on the 0th, 3rd, 6th, 9th, 12th and 15th day of the experiment. The effect was more pronounced in standard (10 mg/kg) group, which shows significance decrease in blood glucose level (*p* < 0.001) from 3rd to 15th day of the experiment.

The methanol extract of *Z. armatum fruit* (Zf) caused the significant (*p* < 0.05) decrease in blood glucose level of normal group of mice on 15th day. Zf proved very effective and decrease the blood glucose level diabetic mice significantly (*p* < 0.001) from 3rd to 15th day of the experiment. The methanol extract of *Z. armatum bark* (Zb) showed no significance activity on normal group of mice. Zb also showed significant ((*p* < 0.001) activity when compared with diabetic control. The methanol extract of *Z. armatum* leaves (Zl) effect was normal on normal group of mice and no significant effect was observed. Zl showed significant activity (p < 0.001) on blood glucose level of the diabetic mice from 3rd to final day of the experiment when compared with diabetic control. The effect of Zl on blood glucose level of diabetic mice was comparable with the standard drug (Glibenclamide). All the results are tabulated in Table 2.

**Table 2:** Effect of extracts of *Zanthoxylum armatum* and standard drug Glibenclamide on glucose level of normal and alloxan induced diabetic mice

Groups	Glucose level (mg/dl)						
	O^th^ day	3rd day	6th day	9th day	12th day	15th day	% variance
Normal control	110.5 ± 14	99.83 ± 17	99.33 ± 18	102.2 ± 28	107.5 ± 3	109.2 ± 2	4.69%
Diabetic control	333.7 ± 1*	344.0 ± 2*	345.3 ± 22*	335.8 ± 40*	358.0 ± 20*	374.7 ± 16*	6.80%
Normal Zf treated	111.2 ± 13	107.5 ± 15	104.5 ± 16	98.67 ± 14	98.33 ± 14	78.83 ± 34**	11.46%
Diabetic Zf treated	334.2 ± 44.6	298.0 ± 1*	255.8 ± 25*	250.0 ± 30*	234.8 ± 2*	215.8 ± 30*	16.48%
Normal Zb. Treated	114.0 ± 15.	109.7 ± 13	112.7 ± 13	101.3 ± 12	94.67 ± 10	94.5 ± 12	8.47%
Diabetic Zb treated	279.8 ± 36	265.8 ± 3*	259.2 ± 38*	252.2 ± 35*	246.3 ± 3*	239.2 ± 33*	5.65%
Normal Zl treated	106.8 ± 17	105.3 ± 15	108.8 ± 17	99.6 ± 14	100.2 ± 11	98.83 ± 14	4.09%
Diabetic Zl treated	325.2 ± 26	234.0 ± 3*	188.8 ± 36*	154.0 ± 23*	147.5 ± 2*	123.7 ± 18*	37.95%
Diabetic Glibenclamide treated	320.7 ± 22	271.5 ± 18*	215.7 ± 18*	154.2 ± 18*	163.0 ± 1*	136.3 ± 17*	34.84%

Data represented as mean ± S.D. values of 6 animals each *p < 0.001, **p < 0.05 (One way ANOVA, Dunnet’s t-test, Graph pad prism software). Normal control was compared with normal control. and extract treated. The Diabetic control was compared with diabetic extract treated and standard

### Effect of different extracts of *Zanthoxylum armatum* on biochemical parameters

#### Hb

The effect of alloxan monohydrate on mice hemoglobin level was significant (*p* < 0.001) and it reduced the hemoglobin level to 6.278 ± 0.45 when compared with normal group (9.453 ± 0.4).

All the normal groups treated with extracts (Zf, Zb and Zl) showed no significance fluctuation of hemoglobin level when compared with the normal control. However, there is a significance (*p* < 0.001) improvement in blood hemoglobin of the diabetic mice treated with the extracts and standard drug (Glibenclamide 10 mg/kg) when compared with diabetic control. All the results are tabulated in Table 3.

**Table 3:** The effect of extracts of *Zanthoxylum armatum* and standard drug Glibenclamide on Hb level of normal and diabetic mice

Group	Hb (g/dL)
Normal	9.453 ± 0.4
Diabetic	6.278 ± 0.45*
Normal Zf treated	9.09 ± 0.84
Diabetic Zf treated	9.608 ± 0.28*
Normal Zb. Treated	9.020 ± 0.85
Diabetic Zb treated	8.26 ± 0.25*
Normal Zl treated	9.17 ± 0.89
Diabetic Zl treated	8.30 ± 0.30*
Diabetic Glibenclamide treated	8.20 ± 0.27*

Data represented as mean ± S.D. values of 6 animals each *p < 0.001 (One way ANOVA, Dunnet’s t-test, Graph pad prism software). Normal control was compared with normal control and extract treated. The Diabetic control was compared with diabetic extract treated and standard

#### Total proteins

The alloxan monohydrate treated diabetic group of mice (diabetic control) showed significance (p < 0.001) decrease in total proteins level when compared with normal control. When observed the effect of all the extracts (Zf, Zb and Zl) and standard drug on normal and diabetic mice, no prominent change in proteins level was noted in any case. All the results are tabulated in Table 4.

**Table 4:** The effect of extracts from *Zanthoxylum armatum* and standard drug Glibenclamide on total protein level of normal and diabetic mice

Group	Total proteins (mg/dL)
Normal	5.367 ± 0.68
Diabetic	4.03 ± 0.51*
Normal Zf treated	5.58 ± 0.66
Diabetic Zf treated	4.13 ± 0.49
Normal Zb. treated	5.00 ± 0.92
Diabetic Zb treated	4.11 ± 0.41
Normal Zl treated	5.51 ± 0.71
Diabetic Zl treated	4.11 ± 0.37
Diabetic Glibenclamide treated	5.53 ± 0.71

Data represented as mean ± S.D. values of 6 animals each *p < 0.001 (One way ANOVA, Dunnet’s t-test, Graph pad prism software). Normal control was compared with normal control and extract treated. The Diabetic control was compared with diabetic extract treated and standard

#### Urea

Alloxan monohydrate treated diabetic mice showed a significance (*p* < 0.001) increase in serum urea level of mice. Normal mice treated with the extracts for 15 days showed no prominent change in urea level when compared with the normal control. When compared with the diabetic control the extracts (Zf, Zb and Zl) treated groups after 15 days of treatment showed a significance (*p* < 0.001) improvement (decrease) of urea level. The standard drug also showed similar results.

#### Creatinine

When compared with the normal group the diabetic control group showed a significant (*p* < 0.05) increase in serum creatinine level. The normal mice groups treated with extracts (Zf, Zb and Zl) showed no prominent effect on serum creatinine level. However, a significance (*p* < 0.05) decrease in serum creatinine level was observed with extracts (Zf and Zb), less significance (*p* < 0.001) with Zl and standard drug glibenclamide when compared with the diabetic control. The results are tabulated in Table 5.

**Table 5:** The effect of extracts of *Zanthoxylum armatum* and standard drug Glibenclamide on serum urea and creatinine level in normal and diabetic mice

Groups	Urea (m mol/L)	Creatinine (μ mol/L)
Normal	5.16 ± 0.42	28.20 ± 1.82
Diabetic	17.70 ± 0.85*	37.38 ± 0.80**
Normal Zf treated	4.88 ± 0.33	29.55 ± 1.04
Diabetic Zf treated	12.10 ± 1.52*	34.30 ± 0.69**
Normal Zb treated	4.71 ± 0.45	29.52 ± 1.06
Diabetic Zb treated	12.95 ± 0.56*	34.5 ± 0.73**
Normal Zl treated	4.13 ± 0.27	29.55 ± 1.01
Diabetic Zl treated	13.70 ± 1.44*	32.03 ± 1.33 *
Diabetic Glibenclamide treated	17.70 ± 0.85*	32.87 ± 2.37*

Data represented as mean ± S.D. values of 6 animals each *p < 0.001, **p < 0.05 (One way ANOVA, Dunnet’s t-test, Graph pad prism software). Normal control was compared with normal control and extract treated. The Diabetic control was compared with diabetic extract treated and standard

#### TC and TG

**Fig. 1:**
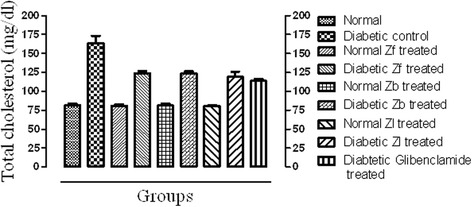
Effect of extracts and standard drug on total cholesterol (mg/dl) level of normal and alloxan monohydrate induced diabetic mice

**Fig. 2:**
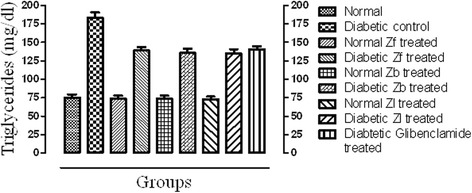
Effect of extracts and standard drug on serum triglycerides (mg/dl) level of normal and alloxan monohydrate induced diabetic mice

There was no significance difference in total cholesterol (TC) and triglyceride (TG) levels when normal control was compared with the normal extract (Zf, Zb and Zl) treated groups. The diabetic control showed hyperlipidemia compared with normal control as indicated by increased level of TC and TG in diabetic mice. The extracts (Zf, Zb and Zl) and standard treated groups significantly decreased the serum level of cholesterol and triglycerides compared to the diabetic control group (*p* < 0.001).The results of hypolipidemic potential of all the plant extracts were comparable to standard drug (Glibenclamide 10 mg/mL) Figs 1 and 2.

#### HDL

**Fig. 3:**
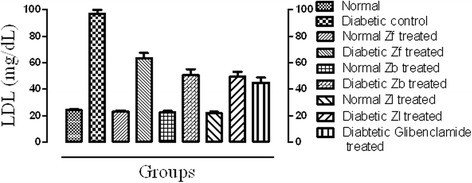
Effect of extracts and standard drug on serum low density lipoproteins (mg/dl) level of normal and alloxan monohydrate induced diabetic mice

Alloxan monohydrate rendered diabetic groups showed a significant (*p* < 0.001) decrease in high density lipoproteins (HDL) serum level compared to the normal control. All the extracts and standard drug in the course of treatment for 15 days showed a significance improvement of HDL level in diabetic mice compared to the diabetic control ((*p* < 0.001) (Fig. 3).

#### LDL

**Fig. 4:**
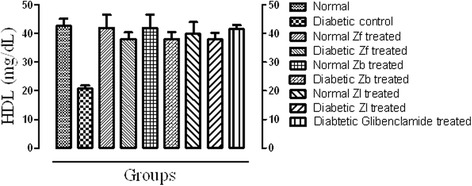
Effect of extracts and standard drug on serum high density lipoproteins (mg/dL) level of normal and alloxan monohydrate induced diabetic mice

In contrast to HLD level, the low density lipoprotein (LDL) was significantly increased in diabetic control compared to normal control. All the extracts and standard drug treated groups showed decreased LDL level significantly (p < 0.001) compared to the diabetic control after 15 days of treatment thereby showing hypolipidemic effect (Fig. 4).

## Discussions

Diabetes has a high prevalence of morbidity and mortality in the world. It is a disease that is not curable but it can be control. A variety of treatments including synthetic drugs, natural medicine and dietary supplements are used to control the diabetes and its related complications.

The use of natural products is very common in the less developed world where these remedies are more accessible and affordable than modern pharmaceuticals. As the research in medicinal plants progressed, more evidences about the effectiveness and safety are available and this is the reason that the use of herbal products as diabetic remedies have increased in the developed world. The incidence of type 2 diabetes mellitus has increased globally which imposed high cost to health services around the world. Due to this fact, there is an increase interest in research in the field of ethnopharmacology for the last two decades and the main focus has been on diabetes. One of the reasons which motivated the research into medicinal plants for diabetic treatment is the lack of effectiveness of the synthetic drug therapy and its consequence of adverse effects [23].

There is a long history of use of medicinal and dietary plants for the treatment of diabetes. Few examples included are, nopal (prickly pear cactus), fenu-greek, karela (bitter melon), gymnema, ginseng, tronadora, chromium, and alpha-lipoic acid. The popularity of these products varies among people of different ethnicities. Nopal is the most commonly used herbal hypoglycemic among persons of Mexican descent. Karela is more commonly used by persons from Asian countries. Some of these agents have gained universal appeal. The studies conducted so far have revealed single or multiple mechanisms of action. Among several of these, high soluble fiber content is a contributing factor. Based on the available evidences, several natural products in common use can lower blood glucose in patients with diabetes [24].

Recently several authors have worked on medicinal plants for their potential role as antidiabetic agents [25–27]. In order to identify the plants with antidiabetic properties various plants have been tested in-vivo using animal models, for example rats, mice, rabbits, against the complications caused by inducers of diabetes, and it has been established that many plants possess the potential to lower the blood glucose levels and besides help in improving other diabetic complications [28]. The antidiabetic effects might be achieved by facilitating insulin release from pancreatic ß-cells, inhibition of glucose absorption in GIT, stimulating glycogenesis in liver and/ or increasing glucose utilization by the body [29].

There are reports of antidiabetic studies on *Zanthoxylum* plants for example *Zanthoxylum zanthoxyloides* leaves exhibits antidiabetic and hypolipidemic effects [30]. Similarly, *Z. armatum* bark showed antidiabetic activity on streptozosin induced diabetes in rats [13].

α-glucosidase is the key enzyme in the digestion of carbohydrates in the surface membranes of intestine. α-glucosidase inhibitors suppress the postprandial hyperglycemia by retarding the liberation of D-glucose of oligosaccharides and disaccharides from dietary complex carbohydrates and therefore delay the glucose absorption [31]. Acarbose, one of such inhibitors are approved in management of type 2 diabetes and for the treatment of obesity [32]. It is necessary to search for more effective and safe α-glucosidase inhibitors from natural materials, in order to develop antidiabetic agents. The extracts of *Z. armatum* showed very significant activity against alpha-glucosidase and all the extract inhibited the enzyme with low concentration comparable with the standard drug acarbose. In vivo studies, the extracts of *Z. armatum* lower the glucose level of alloxan induced diabetes to significant level.

In our studies the methanol extract of *Z. armatum* (fruit, bark and leaves) at a dose of 500 mg/kg showed significant effect on the glucose tolerance of mice and the extracts also showed reduction in the fasting blood glucose levels of the norm glycemic mice, thus revealing the hypoglycemic nature of the extracts. The effect was more pronounced for the methanol extract of *Z. armatum* leaves.

As the insulin is produced in the β -cells of islets of Langerhans. Alloxan monohydrate caused the destruction of β –cells and stops the production of Insulin and results in induction of diabetes. Therefore, in this case the extracts might have produce the hypoglycemic effect by a mechanism not involving insulin [6].

The hypoglycemic effect of the extracts in hyperglycemic mice was studied during 15 days treatment. The difference observed between the initial and final fasting serum glucose levels of extract treated hyperglycemic mice revealed antihyperglycemic effect of the extracts (Zf, Zb and Zl) throughout the period of study. The effect of the extracts was compared to that of reference standard (glibenclamide) and was found to be significant statistically.

It is common observation that the in diabetes mellitus the level of serum lipids are usually high. This elevation can be risk of coronary heart disease. The hyperlipidemia that characterizes the diabetic conditions may be regarded as a result of the uninhibited actions of lipolytic hormones on the fat depots. Therefore, a drug therapy or a dietary provision can reduced the risk of vascular ailments by lowering the serum lipid concentration [33].In the normal conditions of metabolism insulin hydrolyses the triglycerides by activating the enzyme lipoprotein lipase. The deficiency in insulin results in inactivation of these enzymes thereby causing hyper-triglyceridemia. The researchers did report significant changes in lipid abnormalities [34].

The result of this study reveals that the dose of 500 mg/kg of each of Zf, Zb and Zl recovered the level of serum TC and TG in a significant manner (*p* < 0.001) when compared with the diabetic control. The level of LDL, over a period of 15 days was significantly reduced (*p* < 0.001) towards normal as compared with diabetic control. However, the level of cardio protective lipid HDL was improved significantly by all the extracts in diabetic mice. The effect of extracts on HDL level of normal mice was not prominent. This shows hypolipidemic effect of the extracts and the significant reduction of serum lipid levels in diabetic mice after treatment with extracts may be directly attributed to improvements in insulin levels.

Various secondary metabolites isolated recently from medicinal plants have been shown to possess antidiabetic effect for example saponins [35], alkaloids [36] and flavonoids [37] and phenolic compounds can be responsible for the antidiabetic effect by preventing the destruction of β-cells by inhibiting the peroxidation chain reaction [38]. Our analysis of phytochemicals revealed the presence of such constituents in *Z. armatum* [39].

Increased serum levels of urea and creatinine, indicators of impaired renal function [40]. Diabetic control mice showed an increased level of creatinine and urea and this level remained elevated as compared with normal control when treated with extracts and standard over 15 days of treatment and showed little improvement towards normal control. In the present study, total Hb levels in diabetic control were reduced as compared with the normal control, which may be due to the formation of HbA1c (glycated hemoglobin). A previous report has indicated that, in diabetes, protein synthesis is decreased in all tissues, which is due to the relative deficiency of insulin and to depressed synthesis of Hb [41]. In the present study, treatment of diabetic mice with extracts (Zf, Zb and Zl) resulted in a significant increase in Hb levels. This was more prominent in the Zf treated diabetic group. The effect of extracts on test for total proteins indicated a change in serum protein level of diabetic mice and a slight improvement was shown by the groups.

## Conclusion

This study was conducted to explore the potential activity of extracts of plants *Zanthoxylum armatum* in normal and alloxan induced diabetic mice. Although all the extracts presented significant anti-diabetic activity in mice, but, leaves extract of *Z. armatum* was found most potent among the extracts. The extracts also showed improvement in other parameters studied like body weight, serum lipids (triglycerides, cholesterol, HDL and LDL). It is concluded that the methanol extract of *Zanthoxylum armatum* possesses significant antidiabetic activity and appears to be attractive materials for further studies and possible drug development.

## Abbreviations

Hb: hemoglobin
HDL: high density lipoprotein
LDL: low density lipoprotein
TC: total cholesterol
*TG*: triglycerides
***Z. armatum***: *Zanthoxylum armatum*
Zb: bark of *Zanthoxylum armatum*
Zf: fruit of *Zanthoxylum armatum*
Zl: Leaf of *Zanthoxylum armatum*
